# The influence of after-school tutoring on the mental health of middle school students: A mediating effect test based on sleep deprivation and academic performance

**DOI:** 10.1371/journal.pone.0321048

**Published:** 2025-04-30

**Authors:** Lianhua Fan, Ziqi Zhang, Xinyu Li

**Affiliations:** 1 School of Education, Xi’an International Studies University, Xi’an, Shaanxi, China; 2 Department of Education, Shaanxi Normal University, Xi’an, Shaanxi, China; Regional Health Care and Social Agency of Lodi, ITALY

## Abstract

Although Western studies have shown that adolescents’ mental health is intimately linked to after-school tutoring, very few studies have addressed this issue among Chinese adolescents. Herein, this study investigated the effect of after-school tutoring on students’ mental health using an empirical method and a mediation-effect test analysis. Based on 3581 China Education Panel Survey samples, results showed that sleep duration and academic performance act as mediators in the effect of after-school tutoring on students’ mental health. After-school tutoring leads to depressive emotions in students through sleep deprivation, playing a partial mediating effect (*Z* = 2.785, *p* < 0.05). Academic performance is another factor leading to students’ depressive emotions, similarly passing the mediation effect test (*Z* = 2.817, *p* < 0.05). Overall, after-school tutoring affects students’ mental health by depriving them of sleep time, hence influencing their academic performance. We suggest the following. First, the regulation of off-campus tutoring should be strengthened. Second, efforts should be made to alleviate parents’ educational anxiety. Lastly, students’ sleep quality should be ensured, and attention should be paid to monitoring their mental health status.

## Introduction

The marketization of education has driven parents to encourage their children to take advantage of after-school tutoring for good grades and academic success [1]. Noh et al. reported that, in their first year of junior high school, Korean adolescent students participated in extracurricular tutoring for an average of 165.4 minutes each school day [2]. This phenomenon is also observed in other countries such as Japan, Vietnam, and Cambodia [3].^.^However, some studies reported that after-school tutoring increases students’ academic burden, thereby affecting their mental health and even leading to depression (especially in adolescents) [4–7]. Moreover, after-school tutoring triggers anxiety among parents, disrupting the harmonious family atmosphere and disrupting the healthy growth of adolescents.

Zhang et al. have explored the relationship between after-school tutoring and students’ mental health development [8]. For example, among junior high school students aged 12–15 years old, there is a high incidence of depression. Among adolescents, up to 30% prevalence of sleep disorders has been reported [9]. Children with sleep difficulties also experience increased rates of behavioral depression [10]. According to Institute of Psychology of the Chinese Academy of Sciences in 2023, the incidence rate of depression among junior high school students is 30%, which has increased compared to that in 2019 and 2020 [11]. According to the National Depression 2022, there are 95 million patients with depression in China, 30% of which are aged less than 18 years and approximately 50% of which are students [12].

On the one hand, students’ rest time cannot be guaranteed owing to the increasing pressure of schoolwork. On the other hand, millennial parents generally pay more attention to the development of their children, worrying that the next generation will lose the opportunity to learn and enter the elite class. With the popularity of after-school tutoring institutions, parents’ educational anxiety has risen, resulting in negative family atmosphere. Therefore, the influence of after-school tutoring on the mental health of middle school students is an urgent issue.

## Literature review

According to the World Health Organization, approximately 50% of adult mental disorders emerge before the age of 14 years. A random sample survey reported that 46% of students in Shenzhen, China have depressive mood [13]. Moreover, the mental health of junior high school students or adolescents has deteriorated in the past 20 years [14,15]. Therefore, this study aimed to explore the influence of after-school tutoring to students’ mental health from the following dimensions: sleep deprivation, academic performance, and related control variables.

### Relationship between sleep deprivation and mental health

Sleep is influenced by various determinants, such as biological and psychological factors, child characteristics and development, and social and environmental surroundings [16]. Spending too much time on after-school tutoring can lead to insufficient sleep time. An experimental test of one night of sleep deprivation compared to sleeping normally decreased positive mood and increased negative mood [17]. The incidence of sleep problems among Chinese students is nearly 20% [18]. A survey of students from Chinese mainland [19], Korea and Chinese Taipei [20] has reported that after-school tutoring have a negative impact on students’ sleep. The decline in sleep duration increases the risk of mental health problems, such as anxiety, depression, and negative emotions [21]. The cause of mental health problems remains unclear; nevertheless, studies have concluded that it is closely related to sleep [22–24]. The Pittsburgh Sleep Quality Index, which includes sleep efficiency, time to fall asleep, and sleep disorders, has a significantly negative correlation with anxiety and depression [25]. In other words, a higher Pittsburgh Sleep Quality Index score corresponds to a lower quality of sleep.

### Relationship between academic performance and mental health

Extracurricular tutoring can either positively or negatively affect academic performance. In East Asia, private supplementary education is considered a necessity [26]. However, extracurricular tutoring does not automatically improve students’ academic performance, which can be attributed to various factors, such as race and cultural country context [27]. Similarly, the depressive effect of extracurricular tutoring has been mainly reported in students with vulnerable family backgrounds and not in those with wealthy families [28]. However, some researchers believe that this depressive effect has no direct relationship with the family background of students and attribute the reason to the quality of courses and not to the total amount of extracurricular tutoring expenditure [29]. Students from advantageous family backgrounds enroll in expensive extracurricular classes with a high teacher–student ratio and tutoring quality; thus, their learning efficiency also improves, and the effect of sleep deprivation is weakened [30]. Moreover, the goodness and badness of academic performance can affect students’ mental health. Students who perform poorly academically have significantly lower levels of mental health than those who perform well [31].

### Other control variables

The ecosystem theory states that the development of individual psychological behavior is a reflection of the interaction between individual behavior and the social environment [32]. Based on this theory, other control variables from the social environment and individual perspective can be identified. The “Double Reduction Policy,” which refers to a reduction in the amount of school homework and number of off-campus or after-school training programs, can effectively reduce students’ academic burden but not parents’ educational anxiety [33]. Parents’ academic expectations, psychological control, support, or pressure on their children can lead to anxiety among students [34]. Family capital also plays a key role in students’ psychological status [35]. Family social capital affects students’ academic performance, educational investment, and subjective well-being [36]. In addition, a common indicator of a family’s social capital is socioeconomic status. Families with higher socioeconomic status (SES) are more likely to have access to goods and services based on social capital.

The social capital theory also believes that a higher family SES corresponds with higher parents’ participation in their children’s education, as parents value the interaction between home and school, supervise their children’s academics, interact frequently with other parents, and understand new educational developments to promote their children’s academic and physical and mental health development [37]. Family SES is usually calculated by Bradley and Corwyn and includes three indicators, namely income, occupation, and education level [38]. Moreover, parents’ educational level affects students’ relief from bad psychological emotions [3]. Internally, students’ self-efficacy, achievement expectation, and learning motivation also influence their mental health level [39]. Self-efficacy plays a role in depression. Generally, when self-efficacy is low, an individual feels frustrated and depressed [40].

Overall, existing studies exploring the mechanism of depressive mood in adolescents have focused on school factors [41], peer relationship, parent–child relationship [42], and parenting style [43,44]. However, the influence of after-school tutoring on students’ mental health has not yet been investigated. Whether shadow education affects the mental health of secondary school students and its influences remain unknown. Regarding research methods, the existing studies are mainly limited to the correlation analysis between variables or the heterogeneity analysis of mental health problems among different groups; however, the formation mechanism of mental health problems among middle school students has not yet been explored. Thus, in the context of reduced academic workload of Chinese students and the increased prevalence of mental health problems among students, investigating the influence of after-school tutoring on students’ mental health can provide theoretical support for educational reform.

## Methods

### Data sources

According to the research needs, two phases of the large-scale tracking survey project China Education Panel Survey (hereinafter referred to as CEPS) designed and implemented by China Survey and Data Center (NSRC) of Renmin University of China will be selected. The first phase of the project began in July 2013 with a random selection of 28 county-level units (counties, districts, and survey sites) across the country, and 112 schools were randomly selected at the survey sites, and all students in 438 classes were surveyed. The 2013–2014 school year was used as a baseline by CEPS, and the survey subjects were the students in the first year of junior high school (7th grade) and the third grade of junior high school (9th grade), and the 2014–2015 was followed up with students in the first year of junior high school (7th grade) at the baseline survey, and the project included comprehensive information on nearly 20,000 students. The 2013–2014 student baseline data, 2014–2015 parent tracking data and 2014–2015 student tracking data are merged and cleaned, and the illogical, irrational samples and the sample of students which were lost to follow-up in the second phase of the survey are deleted. After organizing, the samples that participated in after-school tutoring occupied 80.16%, and the average time spent in after-school tutoring classes exceeded 5 hours per week. Since the nation has not published relevant policies to supervise after-school tutoring institutions from 2013 to 2014, and the scale of it has expanded at this stage, the data can reflect the real situation of students’ mental health under large-scale academic tutoring, which has a certain value for us to demonstrate the influence mechanism of after-school tutoring on the mental health of secondary school students. In other words, the data at this stage have the explanatory power to explore how after-school tutoring affects the mental health of secondary school students.

### Selection of variables

#### Selection of mediator.

According to previous empirical studies, there is a strong correlation between the duration of after-school tutoring and the generation of students’ depression, but theoretically it is not possible to directly prove the causality between after-school tutoring and depression of adolescents. Therefore, this study tries to explore the path of suppressing after-school tutoring. sleep deprivation and academic performance were selected as mediating variables based on the previous literature review.

a. Sleep deprivation

According to existing research, after-school tutoring will deprive students’ sleep and rest time, and according to the research of the Chinese Sleep Research Association, insufficient sleep time is the main risk factor for health problems, including psychological problems. Sleep deprivation refers to the condition of not getting enough sleep. Convert the average daily sleep duration involved in CEPS into hours, and exclude the unreasonable value of sleep duration that more than 15 hours according to the amount of time a middle school student spends in school each day. The results show that 89.27% of middle school students sleep less than 9 hours. After the “double reduction”, the five management measures(which refers to homework management, sleep management, mobile phone management, reading material management, and physical fitness management) are further introduced, and the sleep management is listed as an important measure to implement the “double reduction policy”, which stipulates that the sleep time of primary school students should not be less than 10 hours, and the sleep time of middle school students should not be less than 9 hours. Thus, the formula for measuring the degree of sleep deprivation is the average sleep duration of less than 9 hours per day. Positive values are sleep deprived and negative values are sleep adequate, the higher the number, the intenser the degree of sleep deprivation.

b. Academic performance

In China, eighth-grade students are mandated to undertake a curriculum that encompasses Ideological and Moral Education, Chinese Language, Mathematics, Foreign Languages, Science (alternatively Physics, Chemistry, and Biology), History and Society (alternatively History and Geography), Physical Education and Health, Art (encompassing Music and Fine Arts), as well as Comprehensive Practical Activities. These subjects are subject to adjustments based on the specific conditions prevalent in diverse regional contexts. Notably, Chinese Language, Mathematics, and Foreign Languages are recognized as the cornerstone disciplines of basic education and serve as pivotal indicators for assessing students’ academic attainment and performance.Academic performance is the main factor affecting the mental health of middle school students. In the fierce competitive environment of middle school, students with good academic performance tend to have strong self-confidence, which weakens the possibility of depression. And the main function of after-school tutoring is to improve students’ academic performance. Therefore, the data of the academic growth part of the student questionnaire and some items in the parent questionnaire of the CEPS questionnaire were reduced dimensionally to synthesize the academic performance variables.For example, “whether students are struggling to learn Chinese (math and English) at school currently” in the self-evaluation scale; “What is the ranking of the child’s current grades in the class” in the parents’ evaluation scale. In order to ensure the subjective and objective consistency of the study, the scores in the student self-evaluation scale and the that in the parents’ scale were combined as the evaluation grade of students’ academic performance.

#### Selection and processing of control variables.

Considering that previous studies have shown that family social background is an important factor that affecting the effect of after-school tutoring, family socioeconomic status (SES). According to the previous literature review, the quality of after-school tutoring may affect students’ academic performance, so the hourly price of after-school tutoring was included as a control variable as a tool to measure the quality of after-school tutoring. Secondly, for middle school students, parenting style is also an important factor affecting students’ mental health. Students’ academic motivation is an important factor influencing learning outcomes, which may in turn affect the model [45]. Therefore, factors such as student gender, parents’ confidence in their children, parents’ expectations regarding their children’s educational level, and parent-child communication have been included. From the students’ perspective, self-efficacy and self-educational expectations are also key factors affecting students’ emotions. From the perspective of students themselves, students’ self-efficacy and self-education expectations are also key factors affecting students’ emotions.

#### Selection and treatment of causal variables.

Depressive mood refers to a state characterized by feelings of sadness, hopelessness, and low energy levels. It can occur independently or as a symptom of depression and is often associated with negative impacts on mental well-being. Both the baseline survey and follow-up survey organized by CEPS involve the mental health status of students. The test contains 10 indicators which measures anxiety or depression, each in the form of a likert variable, where “never” = 1, “rarely” = 2, “sometimes” = 3, “often” = 4, “always” = 5, and the higher the number, the more severe the student’s depression level. The questions in the student questionnaire are based on The Center for Epidemiologic Studies Depression Scale (CES-D), which was developed by Radloff who worked in the United States National Institute of Mental Health in 1977 and it is the official psychometric scale of the United States health agency. The scale is a self-evaluation scale for students, and the mental health condition of junior high school students is measured in five dimensions: upset, depression, unhappiness, meaninglessness, and sadness. Moreover, the self-evaluation depression scale in CEPS has been repeatedly used by multiple research teams and research fields [1,46], and has shown stable psychometric characteristics in many cases.In this study, the scale comprising 10 items was subjected to mean processing, with the mean values ranging from 1 to 5.

In China, the junior high school education consists of two semesters per year, with most schools following a schedule of September to January for the first semester and March to July for the second semester. Adjustments to these semesters may be made in different regions based on local weather conditions, ethnic customs, and other factors. Students attend classes for five days a week during the semester, with Saturdays and Sundays serving as rest days. In eastern China, students typically arrive at school at 8:00 a.m. or earlier, while in western regions, school arrival times are delayed according to local solar time; for instance, in Xinjiang, a western region of China, students usually arrive at school at 10:00 a.m. The Ministry of Education of China stipulates that middle school students’ school hours should not exceed eight hours per day. However, this is often difficult to achieve in practice, and most middle school students spend around ten hours at school each day. Students may have a short break during lunchtime, but this usually does not exceed one hour.“Tutor duration” refers to the total amount of time a student spends on after-school tutoring classes related to their coursework, both during weekdays (Monday through Friday) and weekends.There are two questions that reflect the duration of students’ extracurricular studies. One of it is “The amount of time you usually spend on after-school tutoring classes (related to coursework) each day, from Monday through Friday.” There are six catagories of answer options, recoded as “no” = 0, “less than 1 hour” = 0.5, “1 hour to 2 hours” = 1.5, “2 hours to 3 hours” = 2.5, “3 hours to 4 hours” = 3.5, and “about 4 hours or more” = 6. The second is “On weekends, the amount of time you usually spend on after-school tutoring classes (related to coursework) every day,” and repeat the above. We multiply the tutoring time from Monday to Friday by 5 plus the weekend tutoring time by 2 to get the “extracurricular tutoring time” variable. The larger the value, the more extracurricular tutoring time the individual has invested. And set the tutoring time greater than 80 hours as an unreliable value, if it is higher than 80 hours, the sample will be deleted.(Detailed in Table 1) In China, an excessively long duration of tutoring for students may indicate a deviation from the norm, where education primarily occurs through regular school curricula. This situation is considered atypical. Therefore, the present study focuses exclusively on students actively engaged in compulsory education. To ensure the robustness of our analysis, instances where tutoring exceeds 80 hours are identified as outliers and are excluded from the study’s data set accordingly.

**Table 1:** Summary of Definitions and Descriptions for Variables.

Variable Type	Variable Name	Definitions
**Independent variable**	The duration of tutoring	The duration of tutoring refers to the total amount of time spent in tutoring sessions over a specified period (e.g., weekly or monthly). It is often measured in hours.
**Dependent variable**	Depressive mood	Depressive mood refers to a state characterized by feelings of sadness, loneliness, and a general lack of interest or pleasure in activities.
**Mediating variables**	Sleep deprivation	Sleep deprivation is defined as obtaining less sleep than is required for optimal functioning. The study defines 9 hours as the optimal sleep duration required for optimal functioning among middle school students.。
	Academic Performance	Academic performance is typically measured by grades or standardized test scores and reflects the extent to which a student has achieved educational objectives. This variable is a composite result that integrates students’ self-evaluations of their academic performance (including Chinese, mathematics, and English) and parents’ evaluations of their children’s academic performance.
**Control variables**	Parent’s educational expectations	Parent’s educational expectations refer to the aspirations or beliefs that parents hold regarding the educational achievements of their children. This variable significantly influences the academic pressure that parents exert on middle school students, which in turn affects students’ emotions and daily academic performance.
	Self-efficacy	Self-efficacy is defined as an individual’s belief in their ability to succeed in specific situations or accomplish a task. It plays a crucial role in how goals are approached and has been linked to academic achievement and emotional well-being.
	Self-education expectations	Self-education expectations refer to an individual’s beliefs about their own educational future and capability to succeed academically.
	Family Socioeconomic Status (SES)	Family socioeconomic status is a composite measure that includes family income, education level, and occupational status. SES can have profound effects on access to educational resources and overall academic outcomes.
	Parent-child communication	Parent-child communication involves the quality and frequency of conversations between parents and their children. Effective communication can facilitate emotional support and academic discussions, positively influencing both mood.
	Average price of tutoring classes (hours)	The average price of tutoring classes refers to the typical cost associated with hiring a tutor, measured on an hourly basis. This variable may impact the accessibility of tutoring services and, consequently, student performance outcomes.

### Study model of suppression of after-school tutoring

**Fig 1:**
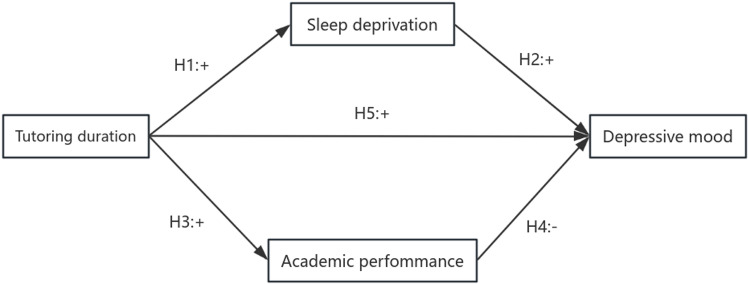
Model of mediating effect (where H1-H5 is the hypothesis of mediating effect and + /- is the positive and negative effect of the influence).

We referenced some literature [47]. This paper adopts a multiple parallel mediation model to study the relationship between the duration of junior high school students’ participation in after-school tutoring and the depressed emotional value of students from grade 7 to grade 8, with sleep deprivation acting as one of the mediating pathways to depression. By depriving students’ sleep time, the length of tutoring leads to the deepening of students’ mental health problems. Academic performance is the second suppression path, with suppression effect, which is a kind of generalized mediating effect. That means good academic performance can alleviate students’ mental health problems to a certain extent, but poor academic performance will strengthen the suppression effect of tutoring time. (Detailed in Fig 1).

Hypothesis 1: Tutorial duration has a positive predictive effect on sleep deprivation

Hypothesis 2: Tutorial duration has a positive predictive effect on depression

Hypothesis 3: Tutorial duration has a positive predictive effect on academic performance

Hypothesis 4: Sleep deprivation has a positive predictive effect on depressive mood

Hypothesis 5: Academic performance has a negative predictive effect on depressive mood

### The selection of research tools and mediating effect test methods

According to the nature of the data and the needs of data consolidation, cleaning and processing, STATA 17.0 is selected as the data processing tool. A more direct method to test the product of coefficients is Sobel mediation analysis test, its testing force is higher than the distribution sequential regression test, and its prerequisite is that the data satisfies the normal distribution. After cleaning, the sample size of the database reaches 3581, and the data distribution can be approximated as normal distribution. According to the Shapiro-Wilk test, the W values for the independent variable, dependent variable, and mediator variable are close to 1, and p > 0.05, indicating that the data are indeed normally distributed. There is a linear correlation between the independent variable and the dependent variable (see Table 2 for details), which meets the requirements for the Sobel test.

**Table 2:** Descriptive Statistics of Variables (*N* = 3581).

		*M*	*SD.*	*Min*	*Max*
**Independent variable**	The duration of tutoring	14.70	12.70	0.00	75.50
**Dependent variable**	Depressive mood	2.13	0.92	1.00	5.00
**Mediating variables**	Sleep deprivation	1.13	1.10	-4.00	5.93
	Academic Performance	6.01	1.32	2.00	9.00
**Control variables**	Parent’s educational expectations	7.21	1.46	1.46	1.00
	Self-efficacy	3.22	0.68	0.68	1.00
	Self-education expectations	2.88	1.06	1.06	1.00
	Family Socioeconomic Status (SES)	-1.15	6.01	6.01	-13.39
	Parent-child communication	2.36	0.51	1.00	3.00
	Average price of tutoring classes (hours)	9.25	17.85	–	222.22

In the dataset, there are 49.10% male samples and 50.90% female samples, with 77.2% being urban students and 22.8% being rural students. All participants are peers in middle school, with ages concentrated between 12 and 14 years old.

## Empirical analysis

### Descriptive statistics of variables

According to the cleaned data, the average weekly tutoring time of junior high school students is 14.70 hours, and the maximum tutoring time is 75.50 hours, with a large group difference and a standard deviation of 12.70. The average depression level of the dependent variable is 2.13, which is at the medium level in the five-point scale. The average duration of sleep deprivation per day is 1.13 hours, the maximum duration of sleep deprivation is 5.93 hours, and the maximum duration of adequate sleep is 4 hours. Academic performance is the sum of parents’ evaluation and students’ self-evaluation, and the average evaluation of students’ academic performance reaches 6, which is above average. And it is consistent with the situation where the subjective evaluation is slightly higher than the reality (in Table 2).

**Fig 2:**
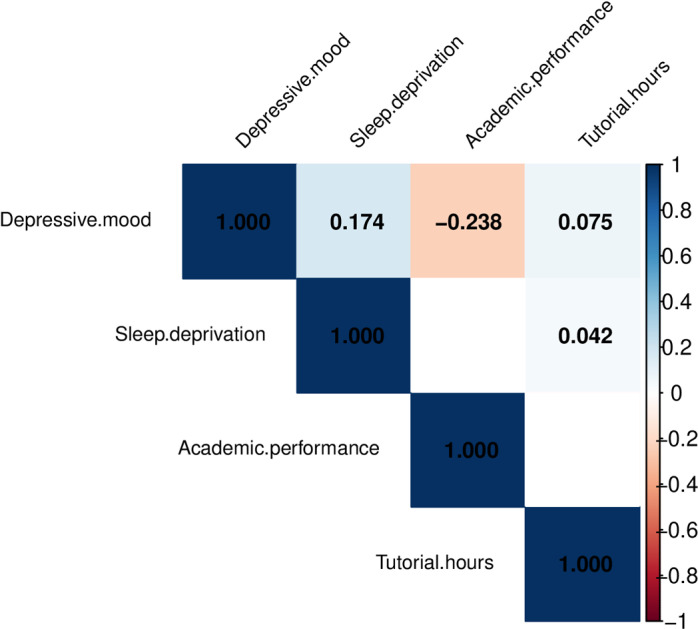
Correlation analysis of variables heatmap.

Correlation test results (in Table 3) showed that depression is significantly correlated with tutoring duration, sleep deprivation and academic performance, and sleep deprivation has the strongest correlation, with correlation coefficient (r) of 0.174. The more sleep deprivation duration, the higher depressive mood. The length of tutoring also has a positive correlation with depression. Depressive mood has negatively correlated with academic performance, which means that the lower the academic performance, the more depression is likely to accumulate (Fig 2, Table 3).

**Table 3:** Correlation Test Table of Dependent Variables, Independent Variables and Mediating Variables.

	Depressive mood	Tutorial hours	Sleep deprivation	Academic performance
**Depressive mood**	1.000			
**Tutorial duration**	0.075^***^	1.000		
**Sleep deprivation**	0.174^***^	0.042 *	1.000	
**Academic performance**	-0.238^***^	-0.024	-0.005	1.000

*** is p < 0.001; ** is p < 0.05; * is p < 0.1.

### Sobel mediating effect test

Sobel’s mediating effect test formula is: $\text{z=}\frac{\text{a }\;\;\times \;\;\text{ b}}{\sqrt{{\text{b}}^{\text{2}}\;\;\;\times \;\;\text{ C+}{\text{a}}^{\text{2}}\;\;\;\times \;\;\text{ d}}}$ a represents the direct effect of the independent variable on the mediating variable, b represents the direct effect of the mediating variable on the dependent variable, c represents the total effect of the independent variable on the dependent variable, and d represents the moderating effect of the independent variable on the mediating variable. The Z-value is used to test the significance of the mediating effect. If the absolute value of the Z-value is greater than the critical value, it indicates that the mediating effect is significant.Using STATA17.0, the sobel mediating effect test is conducted for the two mediating paths, both of paths included all control variables.

#### Path 1: tutoring duration-sleep deprivation-depressive mood.

As shown in Table 4 and Table 5, sleep deprivation passes the mediating effect test as a pathway to depression caused by after-school tutoring, Sobel test (Z = 2.785, *P* = 0.005), indirect effect (a*b) and direct effect (c’) all reached a significant level, indicating that sleep deprivation can be used as a pathway for after-school tutoring to cause students’ depression. And it plays a partial mediating role.

**Table 4:** Sobel Test of Mediating Pathways of Sleep Deprivation.

	Mode1(1):Depressive mood	Model(2):Sleep deprivation	Model(3):Depressive mood
**Sleep deprivation**			0.128***
**The duration of tutoring**	0.005***	0.004***	0.005***
**Parent’s educational expectations**	0.022*	0.069***	0.013
**Self-efficacy**	-0.263***	-0.113***	-0.248***
**SES**	-0.002	0.017***	-0.004*
**Self-education expectations**	0.109***	0.026	0.105***
**Parent-child communication**	-0.101***	0.040	-0.106***
**Average price of tutoring classes (hours)**	0.001	0.003***	0.001
**cons**	24.43***	1.075***	3.092***
**R-squared**	0.133	0.035	0.155
**Prob>F**	0.000	0.000	0.000

*, ** and *** denote signiﬁcance level at the 10%, 5% and 1% levels.

**Table 5:** Sobel Test of Mediating Pathways of Sleep Deprivation.

	Coefficient	Standard Error	*z*	*P > z*
**Sobel**	0.001	0.000	2.785	0.005
**Aroian**	0.001	0.000	2.771	0.006
**Goodman**	0.001	0.000	2.800	0.005
**Coefficient a**	0.004	0.002	2.917	0.004
**Coefficient b**	0.128	0.014	9.373	<0.001
**Indirect effect aXb**	0.001	0.000	2.785	0.005
**Direct effect c’**	0.005	0.001	4.345	0.000

#### Path 2: tutoring duration-academic performance-depressive mood.

As shown in Table 5 and Table 6, academic performance, as a pathway to depressive mood caused by after-school tutoring, passes the mediation effect test, Sobel test (Z = 2.817, P = 0.005), indirect effect (a*b) and direct effect (c ‘) both reaches a significant level, indicating that academic performance can be used as a pathway for after-school tutoring to cause students’ depression. And it plays a partial mediating role (Table 7).

**Table 6:** Statistical table of depressive mood regression analysis in Pathway 2.

	Mode1(1):Depressive mood	Model(2):Academic performance	Model(3):Depressive mood
**Academic performance**			-0.084***
**The duration of tutoring**	0.005***	-0.005***	0.005***
**Parent’s educational expectations**	0.022*	0.248***	0.043***
**Self-efficacy**	-0.263***	0.178***	-0.247***
**SES**	-0.002	0.0194***	-0.001
**Self-education expectations**	0.109***	-0.194***	0.092***
**Parent-child communication**	-0.101***	0.353***	-0.071**
**Average price of tutoring classes (hours)**	0.001	0.001	0.001
**cons**	24.43***	2.132***	3.410***
**R-squared**	0.133	0.33	0.143
**Prob>F**	0.000	0.000	0.000

*, ** and *** denote signiﬁcance level at the 10%, 5% and 1% levels.

**Table 7:** Sobel Test of The Mediating Effect Pathway of Academic Performance.

	Coefficient	Standard Error	*z*	*P > z*
**Sobel**	0.000	0.000	2.817	0.005
**Aroian**	0.000	0.000	2.788	0.005
**Goodman**	0.000	0.000	2.847	0.004
**Coefficient a**	-0.005	0.002	-3.175	0.002
**Coefficient b**	-0.084	0.014	-6.108	<0.001
**Indirect effect aXb**	0.000	0.000	2.817	0.005
**Direct effect c’**	0.005	0.001	4.446	<0.001
**Total effect c**	0.006	0.001	4.765	<0.001

### Path coefficient between variables

**Fig 3:**
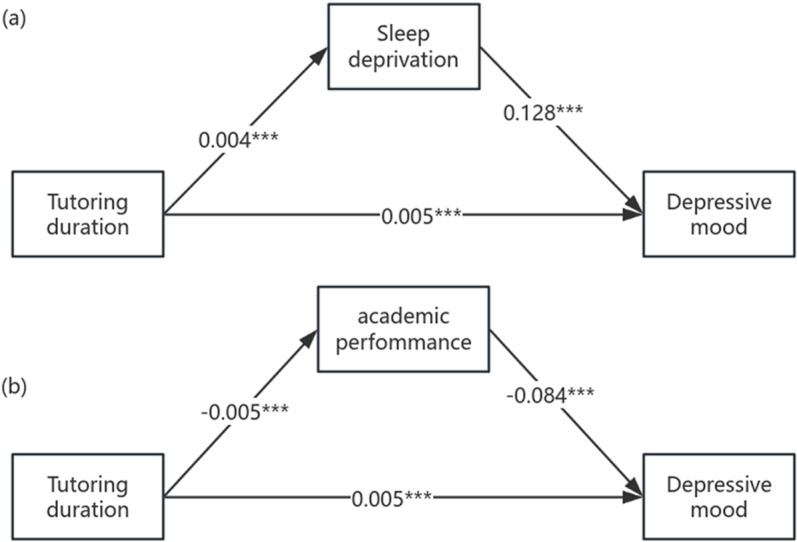
Path coefficients between variables. (*, ** and *** denote signiﬁcance level at the 10%, 5% and 1% levels).

To summarize the regression coefficients for Path 1 and Path 2, you can see in Fig 3.

### Heterogeneity test

Given that the impact of extracurricular tutoring duration on depressive emotions may vary significantly between male and female students, to avoid the heterogeneity of this effect being masked by gender, we conducted a heterogeneity test by gender. As shown in Table 8 below.

**Table 8:** Statistical Table of Heterogeneity Test Results.

Path type		Male			Female		
**Path 1**	Explanatory variable	Mode1(1): Depressive mood	Model(2):Sleep deprivation	Model(3):Depressive mood	Mode1(1):Depressive mood	Model(2):Sleep deprivation	Model(3): Depressive mood
	Sleep deprivation			0.110***			0.141***
	Tutoring duration	0.006***	0.004*	0.005**	0.006***	0.006**	0.005***
	Control variable	Controlled	Controlled	Controlled	Controlled	Controlled	Controlled
	cons	2.968***	1.113***	2.846***	3.478***	0.999***	3.337***
	R-squared	0.121	0.035	0.138	0.147	0.032	0.174
	Prob>F	0.000	0.000	0.000	0.000	0.000	0.000
**Path 2**	Explanatory variable	mode1(1): Depressive mood	Model(2):Academic performance	Model(3):Depressive mood	mode1(1):Depressive mood	Model(2):Academic performance	Model(3): Depressive mood
	Academic performance			-0.088***			-0.094***
	Tutoring duration	0.006***	-0.005**	0.005**	0.006***	-0.003*	0.006***
	Control variable	Controlled	Controlled	Controlled	Controlled	Controlled	Controlled
	cons	2.968***	2.042***	3.148***	3.478***	2.179***	3.685***
	R-squared	0.121	0.293	0.132	0.147	0.363	0.159
	Prob>F	0.000	0.000	0.000	0.000	0.000	0.000

This study conducted a heterogeneity test on the relationship between depressive symptoms, sleep deprivation, and academic performance among males and females. The results indicated that sleep deprivation significantly positively affects depressive symptoms, with both males and females showing this significant relationship. Among males, the coefficients for sleep deprivation were 0.110 and 0.141, suggesting a strong association between sleep deprivation and depressive symptoms. In females, the correlation coefficient was also significant, reaching 0.141. This indicates that the impact of sleep deprivation on depressive symptoms is significant regardless of gender. Furthermore, the duration of tutoring significantly affected depressive symptoms in all models, with coefficients for males ranging from 0.004 to 0.006, and females showing a similar trend, suggesting that tutoring duration may play an important role in moderating depressive symptoms. Overall, the R-squared values for males ranged from 0.035 to 0.138, indicating limited explanatory power of the models for depressive symptoms; whereas the R-squared values for females reached 0.174, indicating a stronger explanatory power for academic performance. In the negative correlation analysis between academic performance and depressive symptoms, the coefficients for academic performance were significant for both males and females, at -0.088 and -0.094, respectively, reflecting the impact of academic pressure on depressive symptoms.

### Robustness test

**Fig 4:**
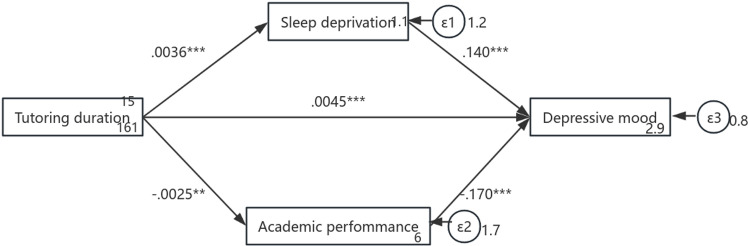
The results of the robustness test coefficient. (*, ** and *** denote signiﬁcance level at the 10%, 5% and 1% levels).

In order to ensure the reliability of the Sobel test method, the study use the structural equation model method to verify the hypothetical model again, and the coefficients of each path of the model are still significant, and the above conclusions of each path (see Fig 4) are still valid. The results of the Sobel test do not have statistical bias.

Based on the statistical data results, it can be concluded that Hypothesis 1, Hypothesis 2, Hypothesis 4, and Hypothesis 5 were accepted, while Hypothesis 3 was rejected.

## Discussion

### After-school tutoring is associated with depression, potentially by affecting students’ sleep patterns

Participation in after-school tutoring can cause depression through sleep deprivation. Long weekly hours of after-school tutoring lead to sleep deprivation, which significantly affects students’ mental health. As previously reported, sleep deprivation can cause depressive mood in students [18]. Furthermore, sleep deprivation due to participation in after-school tutoring is attributed not only to the fact that after-school tutoring takes over rest time, but also to greater academic load, bring high competitive environment out of school, change the self-evaluation of students, infringe on students’ rest time, which aims to achieve the sleep deprivation effect.

### Suppression effect of students’ academic performance disinhibition fails to pass the test

The length of after-school tutoring has a negative predictive effect on students’ academic performance. Therefore, academic performance cannot constitute the suppression effect of after-school tutoring but represents a mediating effect. The suppression effect failing the test may be attributed to the use of 2013–2014 baseline data and 2014–2015 tracking data, and the suppression effect on students’ academic performance exhibits a temporal lag. Moreover, the suppression has a long-term cumulative effect, and data cannot reflect the positive effect of after-school tutoring participation on academic performance in the short term. By contrast, poor academic performance may be a major reason for parents to extend the duration of after-school tutoring. Therefore, the duration of after-school tutoring has a certain negative predictive effect on academic performance; academic performance has a negative predictive effect on depression; and academic performance has a partial mediating effect on the of after-school tutoring.

### Other possible suppression pathways

Because the mediating effect size of the two pathways cannot reach the explanatory power of high probability to explain the depression caused by after-school tutoring, other suppression pathways, such as genetic factors, physical health, students’ family interpersonal relationship, and their relationships and social networks on campus, should be explored [2,10]. Future studies should conduct quasi-experimental research through large-scale data, and more conclusive data on the suppression effect of after-school tutoring can be obtained.

## Implications and suggestions

This study revealed a correlation between extracurricular tutoring and students’ mental health. Based on this conclusion, the following recommendations are proposed.

First, the regulation of off-campus tutoring should be strengthened. If the individual participation’s quality of extracurricular tutoring is low, this delays normal school education and would not be conductive to the academic output of individuals [48]. According to a study by Xue et al., from 2010–2014, there was an increase in the number of students in compulsory education participating in supplemental tutoring; however, the number of those receiving supplemental tutoring declined in 2016 [49]. The government should actively implement the “Double Reduction” policy to address off-campus tutoring. Second, efforts should be made to alleviate parents’ educational anxiety. Zhao et al. pointed out that, in China, parents have high expectations for children to be successful, which lead to great pressure on the children [50]. Thus, parents should adjust their excessive worry regarding entrance examinations and recognize the effect of off-campus tutoring on their children’s mental health. Third, good sleep quality among students should be promoted, and attention should be paid to monitoring their mental health status. According to the BELLA tracking data results in Germany, less than 33% of teenagers with mental health problems have received professional help [51]. Another tracker of middle school students in Japan showed that ensuring sleep quality is an effective measure to prevent mental health problems among adolescents [52]. Ensuring the good quality of students’ sleep is the bottom line of mental health. In addition, the relationship between time allotted for doing homework and academic performance has an inverted “U” curve, showing that declining sleep duration does not lead to higher academic performance [53]. Therefore, sleep quality should be considered a risk factor affecting depression among students, and ways to effectively monitor the duration and quality of students’ sleep should be implemented. Moreover, improving students’ sleep management should be explored.

## Limitations and future prospects

This study had some limitations. First, the studied factors that may influence students’ mental health were limited. Depression may also be related to genetic and physical health factors, especially physiological and hormonal changes occurring after puberty [54], but paucity of data in this area makes it difficult to control in the survey. Second, the definition of sleep deprivation is relatively narrow, failing to consider a broader range of factors, such as sleep quality and the relationship between rest periods and sleep duration. Third, there are limitations inherent to the cross-sectional study design. According to the Chinese Sleep Research Society, sleep problems can lead to changes in mental health development, but mental health conditions can also worsen sleep problems. Therefore, the relationship between the lack of sleep and mental health problems is complex, and there are certain endogenous problems, which require further research. Lastly, owing to the limited data in the database, whether sleep duration is under the influence of drug action could not be clarified.

Moreover, after-school interest class tutoring may occupy students’ sleep time and bring about a suppression effect; however, in data processing, the suppression effect of after-school tutoring time was not significant, and, therefore, it was not included in the discussion of the research results.

Future research on this topic can be optimized as follows. First, the potential factors affect students’ psychological health should be explored, and more relevant variables should be incorporated into the research model for increased explanatory power. Second, there should be stricter control on the sleep deprivation variable; this can be done by using more rigorous methods and collecting more comprehensive data. Third, considering the hysteresis of extracurricular tutoring on students’ psychological health, setting up longer intervals for follow-up surveys is advisable.
